# The impact of long dry periods on the aboveground biomass in a tropical forests: 20 years of monitoring

**DOI:** 10.1186/s13021-020-00147-2

**Published:** 2020-05-30

**Authors:** Milton Serpa de Meira Junior, José Roberto Rodrigues Pinto, Natália Oliveira Ramos, Eder Pereira Miguel, Ricardo de Oliveira Gaspar, Oliver L. Phillips

**Affiliations:** Department of Forest Engineering, University of Brasília, Brasília, Brazil

**Keywords:** Long period drought, Carbon sink, Forest dynamics, Atmospheric carbon

## Abstract

**Background:**

Long-term studies of community and population dynamics indicate that abrupt disturbances often catalyse changes in vegetation and carbon stocks. These disturbances include the opening of clearings, rainfall seasonality, and drought, as well as fire and direct human disturbance. Such events may be super-imposed on longer-term trends in disturbance, such as those associated with climate change (heating, drying), as well as resources. Intact neotropical forests have recently experienced increased drought frequency and fire occurrence, on top of pervasive increases in atmospheric CO_2_ concentrations, but we lack long-term records of responses to such changes especially in the critical transitional areas at the interface of forest and savanna biomes. Here, we present results from 20 years monitoring a valley forest (moist tropical forest outlier) in central Brazil. The forest has experienced multiple drought events and includes plots which have and which have not experienced fire. We focus on how forest structure (stem density and aboveground biomass carbon) and dynamics (stem and biomass mortality and recruitment) have responded to these disturbance regimes.

**Results:**

Overall, the biomass carbon stock increased due to the growth of the trees already present in the forest, without any increase in the overall number of tree stems. Over time, both recruitment and especially mortality of trees tended to increase, and periods of prolonged drought in particular resulted in increased mortality rates of larger trees. This increased mortality was in turn responsible for a decline in aboveground carbon toward the end of the monitoring period.

**Conclusion:**

Prolonged droughts influence the mortality of large trees, leading to a decline in aboveground carbon stocks. Here, and in other neotropical forests, recent droughts are capable of shutting down and reversing biomass carbon sinks. These new results add to evidence that anthropogenic climate changes are already adversely impacting tropical forests.

## Background

Monitoring vegetation changes in natural ecosystems is key to understanding the complexity of ecological processes, including the interactions between species and responses to environmental conditions as they change over time and space [1]. Special attention has been focused on tropical forests due to their ecological importance, characterized by environmental heterogeneity, high biological diversity, and globally significant carbon stocks and dynamics [2–4]. With increasing anthropogenic interference in natural landscapes and global climate change, understanding how forests respond to these changes is becoming increasingly important. The results of such investigations have value in helping to validate model predictions of global change impact on tropical forests [5], and in practical terms can assist in proposing alternative management practices for conservation and forest production that are resilient to environmental changes [6].

The dynamic equilibrium, which is generally described in vegetation monitoring studies, is the result of cyclical fluctuations [7–9]. These fluctuations are characterized by periods of increasing forest density and biomass, and to a lesser extent, a reduction in the number of individuals and biomass. More importantly, all of these features contribute to the maintenance of a stable community structure [3, 9, 10]. Studies on community and population dynamics indicate that both internal and external events are important catalysts of vegetation change. These can be natural; such the opening and regeneration of clearings, rainfall seasonality, or seasonal droughts [11]; or anthropogenic, such with forest fire and felling of the forest [12]. Additionally, global climate change impacts forests [13, 14]. Climate change may promote an increase in tree mortality which in turn can trigger successional processes [15]. This is seen through the establishment and growth abundance of species that have a fast growth rate and intrinsically have a short life cycle, leading to overall reduction in tree longevity and potentially loss of carbon stocks [16]. Increasing tree mortality rates have thus been identified as one of the main threats faced by tropical forests [17] in the face of environmental changes.

The increase in greenhouse gas concentrations is widely acknowledged as the major cause of recent increases in global average temperature [13, 18, 19]. If this current trend continues, future climate changes are likely to result in increases in global average temperature of > 2 ℃, as well as changes in the frequency and severity of extreme droughts and waves of heat [20]. Already, even though many parts of South America have not experienced a decline in total annual precipitation [21], tropical forests are becoming more droughted due to increased intensity of dry seasons [21–23]. In the last two decades, forests in Amazonia and beyond have experienced four multiple severe drought events, during 2005 [24], 2010 [25] and 2016 [26]. In addition, in 2015 and 2016 the El Niño—Southern Oscillation (ENSO) in the tropical Amazon region was at least as strong as the 1997 and 1998 ENSO, itself the largest of the twentieth century [27].

These periods of prolonged drought have affected tropical vegetation, with increases in tree mortality being the most evident factor in all periods [14, 28]. Across Amazonia we know that long-term plots have been experiencing increases in dry season intensity and that this is impacting forest composition [29, 30]. Individual droughts have also negatively impacted biomass, largely through enhanced mortality which is diminishing the size of the forest carbon sink [e.g. 12, 21]. Climate change also has the potential to increase the occurrence, size and intensity of forest fires, and is primarily associated with drier, warmer climates in several regions of the world [31, 32]. Forest fires can cause significant changes in the structure and composition of species in forests, especially in sensitive environments such as tropical forests [33], and changes in forest structure may occur faster than changes in biodiversity [34].

Overall, increased frequency of extreme drought events can cause disruption of large parts of the Amazon forest subject to mortality rates [35]. And when drought events provide conditions for forest fires to occur, degradation in tropical forests tends to be greater [36]. Data from multiple permanent plots in forest ecosystems have been used to show that increases in tree mortality rates can substantially reduce the carbon stock and carbon sink potential of tropical forests [14], but it is far from clear how pervasive these effects are and large areas of tropical forest go essentially unmonitored. A particular gap is the huge area of endangered forests at the transition between the Amazon and adjacent Cerrado in central Brazil. In spite of a few recent studies in eastern end of these systems [37, 38] we know little about how such transition zones—potentially already on the edge climatically due to their marginal status—are faring in the changing climates of the early twenty-first century. In the Amazon-Cerrado transition, several types of forest formations occur [39, 40]. We have been monitoring a moist tropical forest outlier in southern Mato Grosso, central Brazil, for 20 years (1996–2016) using a careful permanent plot methodology with regular reinventories of woody vegetation in a valley forest. These have been subjected to period droughts, and, in some localities, also to recent fire. Now, using these records we here evaluate the structural and dynamic changes of the forest and identify two key questions: First, what is the behaviour of aboveground biomass and the number of stems over 20 years of monitoring? Second, how have forest dynamic processes of productivity, mortality and recruitment changed over time in response to periodic droughts and to fire?

## Methods

In this study, we evaluated the effects of long dry periods on the structure of a tropical forest over 20 years.

### Area of study

The study was carried out in the *Véu de Noiva* Forest Valley (FVVN), located in the *Chapada dos Guimarães* National Park, 15º 24′ 18.80″ S and 55º 49′ 55.35″ W. The FVVN is a fragment of tropical forest, and although located in the Cerrado biome, it is a mix between the gallery forest, which is located in near the water course at the bottom of the valley, and the seasonal forest, which occupies the slope of the valley [41] and is influenced floristically by the Amazon and Atlantic Forest [42].

According to the classification of Köppen, the climate of the region is of the Cw type, which is characterised by a subtropical humid climate and dry winter [43]. The cold and dry periods usually comprise the months of May to September, and the rainy season, October to March. The rainy season contributes approximately 80% of the annual precipitation [44]. Based on data from the INMET Meteorological Database for Teaching and Research (BDMEP) in June 2017, the mean annual rainfall was 1680 mm, with an average temperature of 24.6 °C during the last 20 years [44].

The soil in the FVVN is shallow with rocky outcrops and fairly steep topography [45]. This is because most of the forest covers the slope that was formed just below the cliffs, by the deposition of debris from the erosive process in the valley. Therefore, soils are classified as predominantly Litolics in the sandy phase, or spots of Quartz Sands and Alluvial Soils, which generally occur at the bottom of the valley [45]. A topographic gradient exists within the valley (low, medium and high slope), and Pinto, Oliveira-Filho, and Hay [44] identified five microsites, determined by the topographic position and the type of source rock (phyllite and sandstone). While the structure of the vegetation can differ between them these microsites are distributed equally on both sides of the valley, which allows us to analyze the forest as a whole.

The vegetation in the FVVN is well preserved as it is within an area only prone to natural disturbances, such as opening of clearings by the natural fall of trees [46]. In July 2010, the vegetation was affected by a forest fire [47]. This was the first forest fire registered in the FVVN since the creation of the *Chapada dos Guimarães* National Park in 1989 (see Additional file 1: Figure S1), based onpersonal comments from Park managers, and probably for many decades before, because of the complete absence of fire scars on vegetation.

### Data collection

**Fig. 1:**
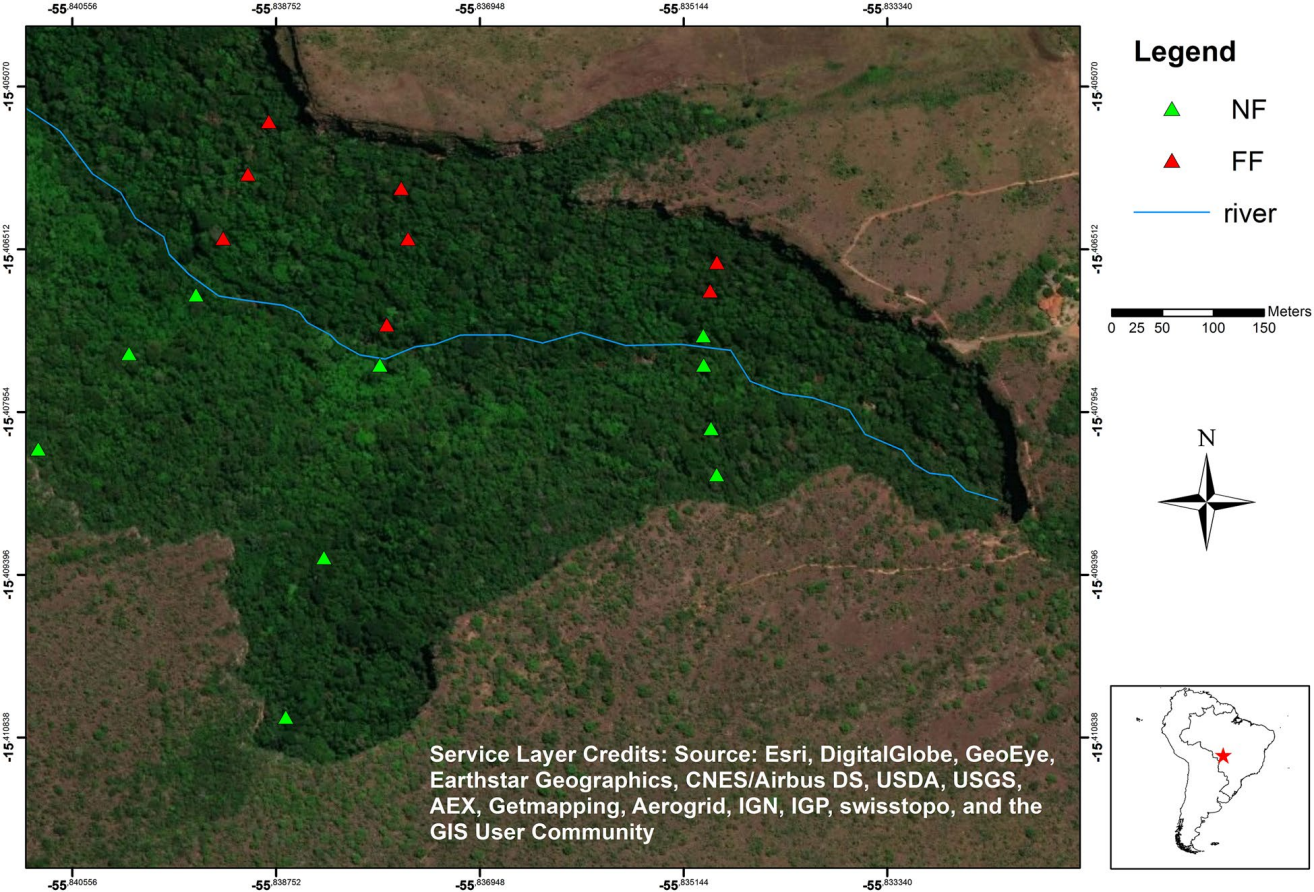
Spatial distribution of plots in the *Véu de Noiva* Forest Valley, *Chapada dos Guimarães* National Park, *Mato Grosso* State, Brazil. Where: sites with forest fire (FF, red) and without forest fire (NF, green) registered in 2010

We sampled the woody vegetation in 18 permanent plots with area of 600 m^2^, totalling 1.08 hectare. This area is systematically distributed into three transects perpendicular to the watercourse, including a total of nine plots of 600 m^2^ on each side of the slope of the valley (Fig. 1 ). The plots were positioned in three topographic sectors: the middle and the top of the slope and close to the water course. The plots positioned in the middle and at the top of the slope are rectangular (20 × 30 m) and the plots positioned at the edge of the stream have a transect shape (10 × 60 m). This form of transect was adopted to better capture the riparian effect of vegetation that occurs close to the watercourse [45].

We sampled all living and remaining tree recorded in previous surveys, and included new individuals (recruits) who met the minimum inclusion criterion: diameter measured at 1.3 m aboveground (diameter-at-breast-height, DBH) greater than 5 cm. The measurements were always taken in the same position, 1.30 m above the ground (DBH). Most trees being small here buttressing at 1.30 m stem height was not an issue. The aluminum identification plates of the individuals were fixed with nails, always respecting the 1.30 m position. Thus, all measurements of the diameter were performed taking as a reference point the position of the tree identification plate. Additionally, to guarantee the measurement of DBH in the same position in all six measurements, we fixed the height of 1.30 m on the shirt of the person responsible for measuring diameter. Finally, to avoid the potential for transcription errors, the field records contained the values of the previous measurements, which facilitated in-the-field checking against the values of the last measurement. Thus, any potential outliers could be immediately identified and checked in the field. To make the reader’s understanding clear, this information is now included in the manuscript.

For the remnants and recruits, we measured the DBH and total height (H). To review and update the names of the species, we referred to the database of the List of Species *Flora do Brasil* [48], which uses the botanical classification system Angiosperm Phylogeny Group—APG IV [49]. Sampling followed the same methodological procedures adopted in the six surveys already carried out at the FVVN, in the years 1996, 1999 [46], 2003 [45], 2006, 2010 [47] and 2016. Plot establishment and protocols are described in detail elsewhere [40, 43, 44].

We directly measured the wood density (WD) of the species that correspond to 80% of all individual trees, in relation to the 2016 census. For the remaining 20% of individuals we used the weighted mean of all other species. To measure WD, we collected a non-destructive sample of wood extracted from the trunk of trees, with the aid of the increase factor, using a Thread Increment Borers (Pressler core borer) [50], which is a minimally invasive method for sampling wood. To obtain the wood samples, we randomly selected five tree per species with selection probability being proportional to the diametric range of the species. For the determination of WD, we followed the methodology proposed by Smith [51]. We separated the plots into two sites conditions, according to the record of occurrence of forest fire recorded in September 2010: (1) site with forest fire (FF) registry, with eight plots; and (2) site without forest fire (NF) registry, with 10 plots.

We sought information about the long periods of drought that occur in the study region. For this, we measure self-calibrating palmer drought severity index (scPDSI) [52]. The scPDSI is calculated from time series of precipitation and temperature obtained by data every 0.5º for latitude and longitude in homepage climexp.knmi.nl.

### Data analysis

To calculate aboveground biomass we adopted the equation proposed by Chave et al. [53], considered as the best fit equation for data for forest formations in the tropical region and used the BIOMASS package [54] in software R [55]

$$AGB = 0.0673 \times \left( {WD \times H \times DBH^{2} } \right)^{0.976}$$

AGB = aboveground green biomass (kg), WD = woody density (g.cm^−3^), H = tree height (m), DBH = diameter measured at 1.3 m aboveground (cm).

To determine the changes in density of tree within the woody community assembly, we used the equation of Sheil et al. [56], which considers the mortality rate based on the initial number of trees, and the recruitment rate based on the final number of trees. As the time interval between measurements was not constant, we applied the correction factor proposed by Lewis et al. [57] ($\lambda_{corr}$). This corrected small biases caused by the influence of differing census intervals and allowed us to estimate the dynamics of the woody community assembly. We thus calculated the mean annual rates of mortality (M), and recruitment (R):

$$M = 100 \times \left[ {1 - \left( {N_{0} - N_{m} /N_{0} } \right)^{1/t} } \right]$$

$$R = 100 \times \left[ {1 - \left( {1 - (N_{r} /N_{t} } \right)^{1/t} } \right]$$.

$$\lambda_{corr} = \lambda \times t^{0,08}$$

where *t *= time between monitoring; N_0_ = initial number of individuals; N_m_ = dead number of individuals; N_r_ = number of recruits; N_t_ = final number of trees; and $\lambda_{corr}$ = crected rate of mortality or recruitment, as suggested by Lewis et al. [58].

Wferred to the sequential intervals between the measurements as1 (1996–1999), I2 (1999–2003), I3 (2003–2006), I4 (2006–2010) and I5 (2010–2016). We classified the tree trunks into three categories: (A) small (between 5 and 10 cm DBH), (B) medium (between 10 and 35 cm DBH), and (C) large (greater than 35 cm DBH) [58]. We used these categories to arrive at a better understanding of the changes in the structure of the woody community assembly.

We calculated the productivity in aboveground biomass (PB) by summing the aboveground biomass of recruiting individuals, and the growth of the surviving trees, relative to time [14] $PB = \sum B_{i} + \sum B_{j} - B_{j - 1}$ where: Bi = Where: B_i_ = biomass of recruits tree, B_j_ = subsequent monitoring biomass for tree; B_j-1_ = previous monitoring biomass for tree. We measured net biomass change (NBC) between each interval as the difference between the later and the previous biomass totals $, {\text{i}}.{\text{e}}. NBC = B_{n} - B_{m}$, where: B_n_ = total biomass subsequent monitoring biomass; B_m_ = total biomass previous monitoring.

To evaluate the effect of forest fire time and occurrence of the aboveground biomass (AGB) and number of stems (NS) variables, we used the Generalized Estimating Equation (GEE) [57, 58], with the log binding function and the Gamma distribution for AGB, and Poisson for NS. This method of adjustment has an advantage, as it evaluates the temporal autocorrelation that incorporates the correlation structure between observations within the plots [58]. To evaluate the relationship between the predictor variables (AGB, NS, M) and dependent variables (time and forest fire), we performed the modified Wald chi-test [59]. When effects of time, forest fire or interaction between these were apparent a Bonferroni post hoc test was used to assess significance [60]. We performed the analysis using SPSS version 24 [61], and adopted α < 0.05 as the significance level for all analyses.

## Results

**Fig. 2:**
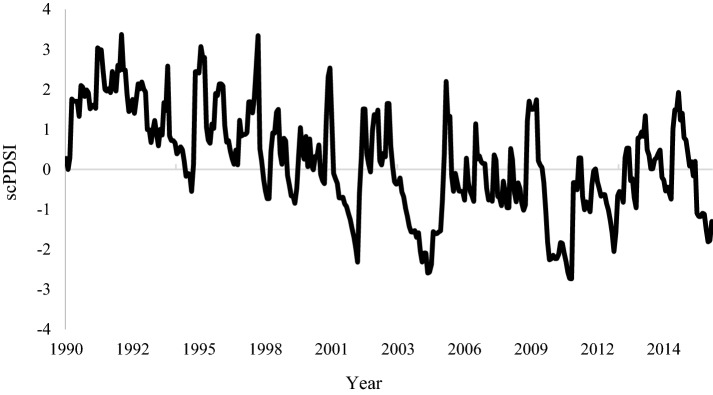
Calibrated drought severity index (scPDSI) for the region of *Véu de Noiva* Forest of Valley, located in the *Chapada dos Guimarães* National Park, Brazil. With emphasis on prolonged periods of drought in the years 2002, 2005, 2010 and 2016

We observed a trend toward increasing drought severity over the years, with four major drought events during the vegetation monitoring: 2002, 2005, 2010 and 2016 (Fig. 2). The 2010 drought period was the most severe in the whole period, coinciding with the forest fire in region FVVN.

Descriptive information on the number of stems, basal area, AGB, mortality rates, recruitment, loss and gain, and the annual periodic increase is presented in Additional file 1: Table S1. We observed that the time effect had a significant influence on mortality and recruitment rates, i.e. that rates differed significantly between the intervals (Additional file 1: Table S2). However, only the mortality rate and AGB differed between NF and FF sites (X^2^w = 11.793; p = 0.01).

**Fig. 3:**
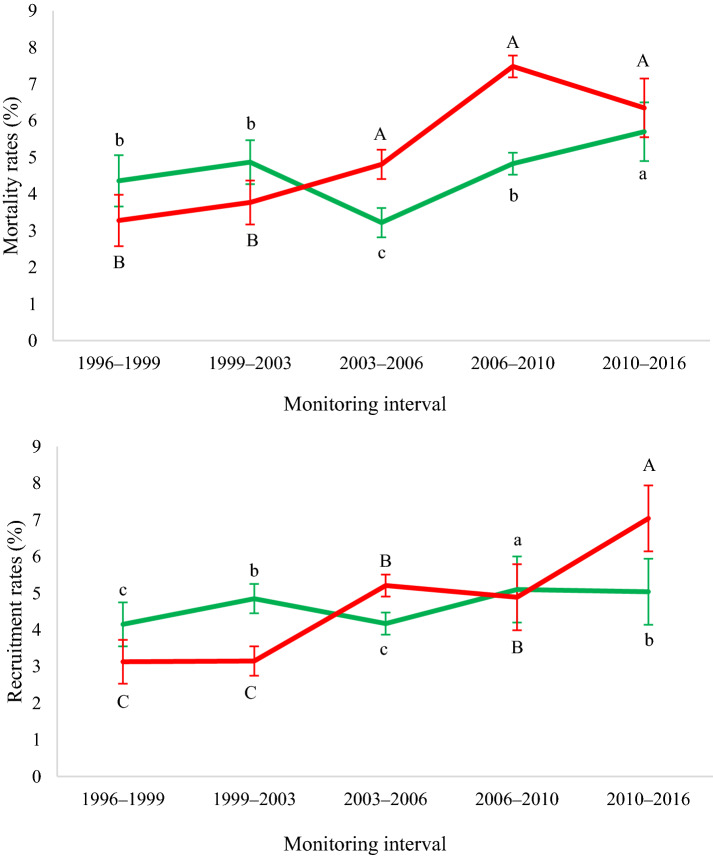
Annual mortality (above) and recruitment rates (below) of individual trees, shown separately for sites which burned in 2010 (red lines) and those which did not (green lines), over 20 years in the Véu de Noiva Forest Valley, Chapada dos Guimarães National Park, Brazil. Equal letters indicate no significant difference among-intervals (Bonferroni post hoc test, α < 0.05). Capital letters are used to indicate differences between monitoring intervals for burned forest, lowercase letters to indicate differences between the monitoring intervals in unburned forests. Error bars represent standard deviation of data

In the NF site, mortality rates tended to increase over time, with little variation in the first measurement interval, a significant reduction in I3 (2003–2006) and a greater increase by I5 (2010–2016), which experienced the highest mortality rate (Fig. 3 and Additional file 1: Table S1). Recruitment rates presented oscillatory behavior, with lower values in the first measurement intervals and significant increase in the latter. In site FF, the lowest mortality rates were recorded early on, in I1 (1996–1999) and I2 (1999–2002), and increased significantly in subsequent intervals, with the highest values recorded during I4 (2006–2010) (Fig. 3 and Additional file 1: Table S1). Recruitment rates here also showed a tendency to increase over the evaluated intervals, with significant differences between evaluated periods and exceptionally high recruitment during I5 (2010–2016).

**Fig. 4:**
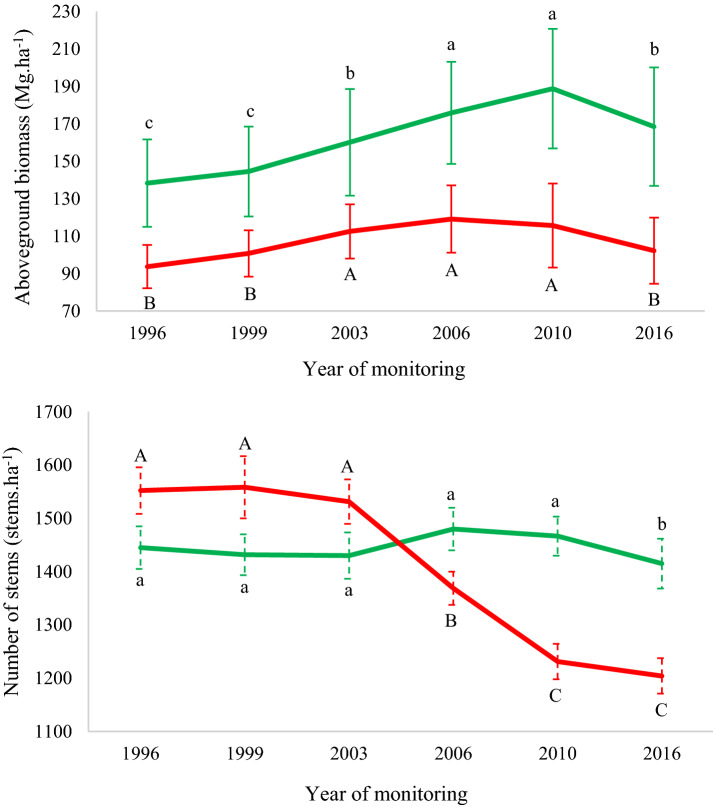
Variation in AGB stock and the number of stems over 20 years in the Véu de Noiva Forest of Valle, Chapada dos Guimarães National Park, Brazil. Green lines represent the site without fire (NF), red lines the site with forest fire (FF) in 2010. Equal letters indicate no significant difference among-intervals (Bonferroni post hoc test, α < 0.05). Capital letters are used to indicate differences between monitoring intervals for burned forest, lowercase letters to indicate differences between the monitoring intervals in unburned forests. Error bars represent standard deviation of data

We observed that the effect of time and sites had a significant influence on AGB (Fig. 4 and Additional file 1: Table S2). For NF, time was the only factor that significantly influenced AGB (Additional file 1: Table S3). At the NF site, AGB increased until 2010, after which, there was a significant reduction, with the value approaching that recorded in 2003, 13 years earlier, but still greater than when we began monitoring it (Fig. 4 and Additional file 1: Table S1). The FF site experienced net AGB gains until the 2006 census. In 2010, the year of the forest fire, there was a reduction in AGB; however, this difference was not significant compared to that of the previous year. By the time of the 2016 census, AGB declined significantly relative to previous years and was similar to values recorded in 1999 (Fig. 4 and Additional file 1: Table S1). Both sites presented reduction in number of steams overtime, significant from 2006 in FF site and from 2016 in NF site, with greater intensity reduction in the FF site (Fig. 4 and Additional file 1: Table S1).

**Fig. 5:**
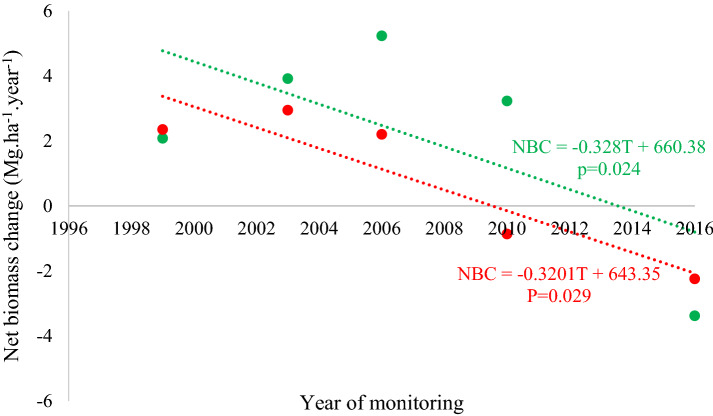
Net biomass change (Mg.ha^−1^.year^−1^) in sites without (green) and with forest fire (red) in 2010, measured since 1996 in the *Véu de Noiva* Forest of Valle, *Chapada dos Guimarães* National Park, Brazil. Note that x-axis indicates the census date at which each monitoring period ended. Dotted lines represent the linear trend in net biomass change rates

We observed that although biomass has tended to increase overall since the start of monitoring, the positive net biomass changes (NBC) from one census to another have declined over time in both sites (Fig. 5). Since 1996, until 2003 in the FF site and until 2006 in the NF site, there was an increase in NBC. Both sites experienced sharp NBC declines since then, although both still ended with higher total biomass than at the start.

**Fig. 6:**
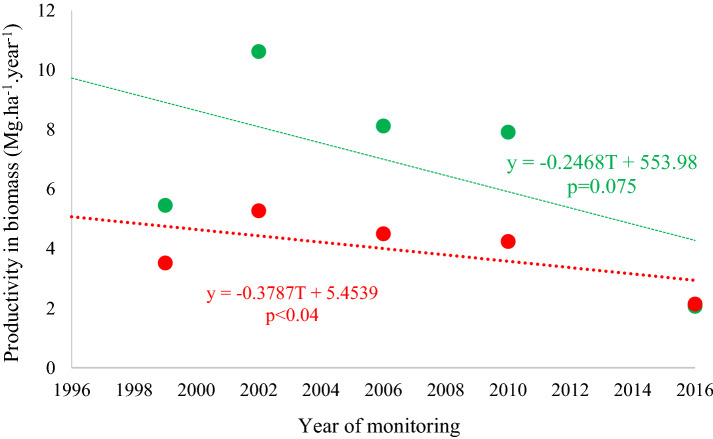
Productivity of above-ground biomass (Mg.ha^−1^.year^−1^) in sites without (green) and with forest fire (red) in 2010, measured since 1996 in the *Véu de Noiva* Forest of Valle, *Chapada dos Guimarães* National Park, Brazil. Dotted lines represent linear trends in biomass productivity

Rates of productivity in aboveground biomass (PB) showed a behaviour similar to that registered for NBC, with substantial increases between 1999 and 2002, and a tendency to decline subsequently (Fig. 6). Overall, the tendency was for biomass productivity to decrease over time as a result of particularly low values in both forests during the final census interval.

**Fig. 7:**
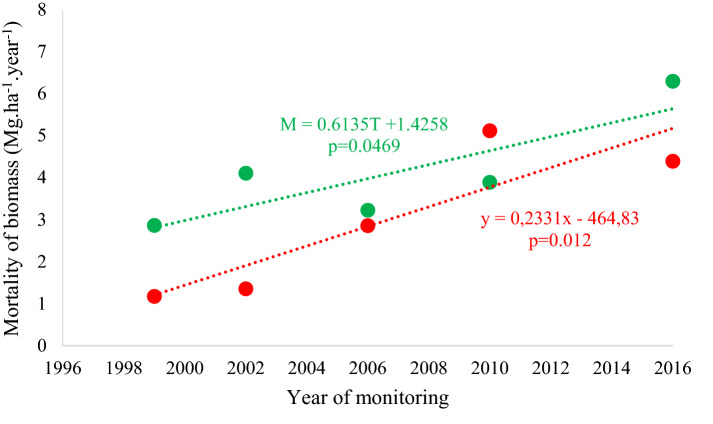
Mortality of biomass by site: without occurrence of the forest fire of 2010 (green) and with the occurrence of the 2010 forest fire (red), registered over 20 years in the *Véu de Noiva* Forest of Valle, *Chapada dos Guimarães* National Park, Brazil. Dotted lines represent linear trends in biomass productivity

**Fig. 8:**
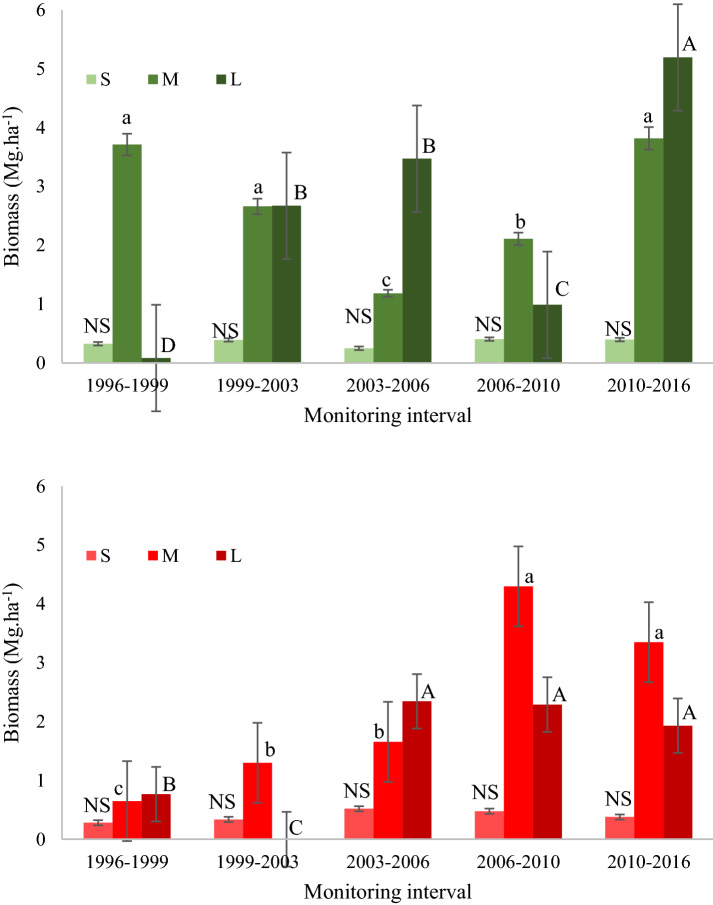
Mortality in biomass aboveground separated in the size classes: Small (S*—*up to 10 cm DBH); Medium (M*—*from 10.1 to 35 cm of DBH) and Large (L*—*greater than 35 cm of DBH), in the sites: without occurrence of forest fire (green scale) and with the fire in 2010 (red scale) registered over 20 years in the *Véu de Noiva* Forest of Valle, *Chapada dos Guimarães* National Park, Brazil. Equal letters indicate no significant difference among-intervals. Capital letters are used to indicate differences between monitoring intervals for the Large size-class, lowercase letters to indicate differences between the monitoring intervals for the Medium size-class. In the Small class mortality rates did not differ significantly (NS) between intervals at both sites (Bonferroni post hoc test, α < 0.05)

Above-ground mortality tended to increase over time. This trend was observed at both the sites (Fig. 7), due to the increase in mortality of medium and large trees (Fig. 8), which even though comprising relatively few individuals, in terms of biomass impact on the forest structure.

## Discussion

We monitored a tropical forest for 20 years in order to evaluate the long-term dynamics of a moist forest outlier at the southern fringes of Amazonia and to explore influence of periods of prolonged drought on the woody vegetation. We observed four periods of drought during the 20 years of monitoring that are in agreement with the information reported by other authors [15]. In the last two decades, Brazil has experienced severe large-scale drought events, during 2005 [24], 2010 [25] and 2016 [26]. Our results show, first of all, that this is intrinsically a very dynamic forest, with high rates of mortality and recruitment even in the site which wasn’t burned and even in those intervals without major droughts. These measurements thus extend and confirm the earlier conclusion drawn from sites further east of here that the southern borders of Amazonia contain some of the most dynamic of all tropical forests—these are truly ‘hyperdynamic’ in terms of stem turnover rates [37] with some of the fastest tree turnover rates recorded anywhere in the tropics.

We noted that in addition to having remarkably high background values of between 3 and 5% each year, mortality rates have also been increasing in the woody vegetation of our study site. These occurred in both the burned and unburned forests. An increase in tree mortality has long been observed generally in many tropical forests [14, 62, 63], including in Brazil [64]. However, the ultimate drivers and proximal mechanisms responsible for this increasing tree mortality remain unknown [15]. Tree mortality often represents a low magnitude disturbance to the forest structure, helping to trigger local processes of forest succession [65, 66], promoting the opening of canopy space which enables increased recruitment [12, 67, 68]. The trend of increasing rates of mortality and recruitment over time has been more pronounced in periods of more prolonged droughts in the tropical region [15]. However, in the site with forest fire, the availability of new space did not result in higher recruitment rates than mortality, which resulted in a net reduction in the number of stems. We can discount stochastic flooding events as a confounding driver of change here, since over the 20 years of study, we have not registered any flooding process in the sampling units, including those positioned near the watercourse, and did not observe any physical consequences of over-banking such as sediment deposition or erosion.

In addition to the long-term increase across out study landscape, our data suggest that mortality rates can also reflect interactions between and forest fire (‘cascade effect’). Thus, during periods of prolonged droughts [69], mortality itself increases the probability of subsequent tree death by opening up the canopy, with environmental changes within the forest favouring the occurrence of fires [31, 32]. The opening in the canopy favours the entry of more light inside the forest, with leaves and other combustible material both drying and increasing in abundance [31, 32]. This drought-fire interaction has been identified elsewhere as responsible for tree mortality in some tropical forests [36]. In part because of this potential for positive feedback, researchers have focused on the impacts of extreme droughts and climate change on tropical forests [27]. Climate change drivers are consistent with the overall increase in mortality rates of tropical forests in general, and especially with the specific patterns of increased mortality of large trees [68] and the fact that the floristic composition of extensive regions of tropical forests has slowly changed to favour those species which have greater resistance to drought [31, 69]. If severe drought events continue to increase mortality rates in tropical forests a more open forest structure could result, which provides conditions for more frequent occurrence of forest fires.

In our forests, the stock of biomass (and therefore carbon, which in these forests is equivalent to AGB × 0.47 [70] generally increased until the most severe drought and the burning of some of our plots (in 2010). A general increase in tropical forest biomass has also been reported widely (in Amazonia, Africa, and Southeast Asia [29, 71, 72]. Indeed the vegetation of the whole terrestrial surface has acted as a strong carbon sink in recent decades, with a substantial fraction of this sink probably located in the tropics, particularly in the Amazon [73]. Overall, structurally intact tropical forests were responsible for half of global terrestrial carbon uptake between 1990 and 2007, so removing ~ 15% of anthropogenic CO_2_ emissions [21, 73]. This widespread increase in tropical forest biomass is often interpreted as a response to the increase accumulation in atmospheric CO_2_ [73, 74], which over long-time scales should favour an increase of productivity biomass in conserved tropical forests [75]. In our site, the increase of biomass reflects the growth of trees already present in the area, since the number of stems did not differ notably over time, a pattern that appears to hold more generally across Amazonia too [14, 70].

In Chapada dos Guimarães National Park, net biomass change during monitoring intervals was strongly positive early on, but from 2006, 1 year after the first major drought of the twenty-first century [24], and became negative by the time of the 2010 drought—a pattern remarkably consistent with the larger South American trend based on an independent dataset [14, 15, 28]. Although tropical forests remain carbon sinks, their capacity to absorb atmospheric CO_2_ appears to be declining [14]. One of the causes of this reduction is climate extremes, which exert a strong effect on biomass when evaluated on a short time scale [75, 76]. Indeed, the sensitivity of tropical forests to environmental changes, especially drought, has already been documented by observational data from permanent plots networks, flux towers, remote sensing and greenhouse gas measurements [29, 77]. Thus, the influence of current climate change—as well of course as more direct human intervention—is significantly impacting the carbon balance of the tropical land surface.

Long periods of drought seem to reduce primary productivity, number of trees, biomass production and increase tree mortality, especially when forest fires occur. We observed that these characteristics began in 2006 and remained during the monitoring period of 2010 (year of the forest fire) and 2016. During these periods, prolonged drought events were also recorded [24–26]. Even in our plots that were not affected by the forest fire, we observed similar behaviors of reduction in biomass production, recruitment and increased mortality. The occurrence of fire in tropical forests is one of the consequences of periods of drought [78] and serves as a catalyst for the reduction of accumulated biomass and productivity, and increase mortality. During years of severe drought, forest fires in the Amazon are typically destructive, killing up to 64% of trees where they occur [79]. Overall, drought periods can significantly affect the structure of the woody vegetation, and especially so when there are fires.

As well as impacts on biomass carbon balance in general, droughts in tropical and temperate forests frequently have greatest impact on larger trees [56]. In our study, large trees experienced increasing mortality, and this is associated with prolonged drought events. The main hypotheses to explain the mortality of trees with drought events invoke either hydraulic failures or carbon ‘starvation’ [56, 64]. Hydraulic failure risks increase in proportion to tree height and tree crown exposure to light and heating, so they are more intensely experienced by larger trees [64]. The hydraulic and carbon balance risks may be associated—in response to the water deficit provided by drought events, and potentially therefore to avoid the risk of hydraulic failure, plants close stomata in their leaves, but in this process the tree may suffer from carbon deficiency and “starve to death” [80]. Regardless, drought-driven mortality of large trees in forests initiates processes of local succession, with the opening of the canopy and the entrance of light [2, 3, 7], and so can increase the temperature of the local microclimate [81], resulting in an even more drought-sensitive environment more prone to forest fire.

Potentially cyclical fluctuations between periods of disturbance and forest reconstruction can trigger longer-term recurrent outcomes, as large trees tend to suffer more from drought than smaller trees [68]. Long-term tropical forest records, including ours, are consistent with greater growth due to increased atmospheric CO_2_ and this fertilization-induced stimulated especially of the growth of larger trees. These, in turn, are precisely those which experience the highest mortality rates during periods of drought, and so a loss of biomass stock. Thus, even without the aggravating effects of fire, contemporary, twenty-first century tropical forests may be especially susceptible to prolonged drought periods, leading to deeper changes in the structure and functioning of these ecosystems than was previously the case.

Lastly, we note that long-term research is clearly crucial to understand and measure the effects of natural and anthropogenic changes on forests. However, these studies are particularly demanding, requiring persistence often across generations of academic careers, and a great deal of standardization and data collection in the field. The present study was no exception. It required 20 years of monitoring as well as considerable financial support and the involvement more than a dozen different graduate students, three master’s dissertations and two doctoral theses. More sustained, long-term initiatives like these are urgently needed so that the effects of climate change on tropical forest formations can be assessed accurately.

## Conclusions

In summary, here we have shown for the first time that moist forest outliers (e.g. valley forest) in central South America are vulnerable to climate change as it reduces the number of stems and increases the mortality of large trees in the forest. Our result extends the conclusion that many Amazonian forests and forests in the Amazon-Cerrado transition are being impacted by anthropogenic climate change as it reduces forest biomass stock. Although valley forests have been thought to be at a lower risk of direct climate impact due to the peculiar relief condition in which they occur. Climatic changes appear to make vegetation more susceptible to forest fires, which compromises the functioning of the forest, the provision of ecosystem services such as carbon sinks, and the maintenance of biodiversity. Overall, our results from a moist tropical forest outlier (valley forest) reinforce the findings from other neotropical forests that recent droughts have shut down and potentially even reversed a long-term biomass carbon sink. Anthropogenic climate changes are already adversely impacting tropical forests.

## Supplementary information

**Additional file 1.** Additional tables and figures.

## Data Availability

Additional data are available in the supplementary information. The data collected in the field can be obtained from the correspondence author.

## Abbreviations

FVVN: *Véu de Noiva* Forest Valley
DBH: Diameter at breast height
H: Total height H
WD: Wood density
FF: Site with forest fire
NF: Site without forest fire
PB: Productivity in biomass aboveground
NBC: Net biomass change
AGB: Aboveground biomass
NS: Number of stems
GEE: Generalized Estimating Equation
