# Correction to: The impact of long dry periods on the aboveground biomass in a tropical forest: 20 years of monitoring

**DOI:** 10.1186/s13021-020-00149-0

**Published:** 2020-07-10

**Authors:** Milton Serpa de Meira Junior, José Roberto Rodrigues Pinto, Natália Oliveira Ramos, Eder Pereira Miguel, Ricardo de Oliveira Gaspar, Oliver L. Phillips

**Affiliations:** Department of Forest Engineering, University of Brasília, Brasília, Brazil

## Correction to: Carbon Balance Manage (2020) 50:12 10.1186/s13021-020-00147-2

Following publication of the original article [1], the authors noticed an error in the article title which was published incorrectly. The correct title is given below: “The impact of long dry periods on the aboveground biomass in a tropical forest: 20 years of monitoring”.

The article title has been updated above and the original article [1] has been corrected.
