# Supplementary material for: The impact of long dry periods on the aboveground biomass in a tropical forests: 20 years of monitoring
Source: Carbon Balance Manag. 2020 May 30;15:12. doi: 10.1186/s13021-020-00147-2 (PMC7261387; doi:10.1186/s13021-020-00147-2)
Supplement: Supplementary file 1 — Additional file 1. Additional tables and figures. [file 13021_2020_147_MOESM1_ESM.docx]

**Supplementary Material**

**Table S1.** Parameters descriptors of the tree vegetation structure (DBH > 5 cm) sampled in the *Véu de Noiva* Forest Valley, located in the *Chapada dos Guimarães* National Park, Mato Grosso State, Brazil. NS= number of stems; BA = basal area, AGB = Above-ground biomass; M = mortality rate in stems; R = recruitment rate in stems; L = rate of loss in basal area; G = rate of gain in basal area and AIP = annual increment periodic.

| **Ano** | **Site** | **NS** | **BA** | **AGB** | **M** | **R** | **L** | **G** | **AIP** |
| --- | --- | --- | --- | --- | --- | --- | --- | --- | --- |
|  |  | **Stems.ha^-1^** | **m².ha^-1^** | **ton.ha^-1^** | **%.year^-1^** | **%.year^-1^** | **%.year^-1^** | **%.year^-1^** | **cm.year^-1^** |
| 1996 | Fire | 1,547 | 19.014 | 93.66 |  | - |  |  |  |
|  | No Fire | 1,448 | 27.480 | 138.24 |  |  |  |  |  |
|  | Total | 1,492 | 23.717 | 118.42 |  |  |  |  |  |
| 1999 | Fire | 1,604 | 19.637 | 100.71 | 3.28 | 3.31 | 1.78 | 2.65 | 0.165 |
|  | No Fire | 1,395 | 27.403 | 144.47 | 4.36 | 4.25 | 2.17 | 2.13 | 0.162 |
|  | Total | 1,487 | 23.951 | 125.02 | 3.86 | 3.80 | 2.15 | 2.41 | 0.164 |
| 2002 | Fire | 1,585 | 20.751 | 112.48 | 3.77 | 3.19 | 1.50 | 2.44 | 0.142 |
|  | No Fire | 1,386 | 27.674 | 160.11 | 4.87 | 5.01 | 2.38 | 2.81 | 0.167 |
|  | Total | 1,475 | 24.597 | 138.94 | 4.34 | 4.12 | 2.18 | 2.80 | 0.155 |
| 2006 | Fire | 1,418 | 21.169 | 119.08 | 4.82 | 3.23 | 4.48 | 4.43 | 0.274 |
|  | No Fire | 1,440 | 28.433 | 175.81 | 3.23 | 4.06 | 2.40 | 3.71 | 0.268 |
|  | Total | 1,430 | 25.205 | 150.60 | 4.57 | 3.75 | 3.37 | 4.15 | 0.271 |
| 2010 | Fire | 1,258 | 20.177 | 115.62 | 7.49 | 5.10 | 5.37 | 3.27 | 0.209 |
|  | No Fire | 1,445 | 28.213 | 188.70 | 4.83 | 5.14 | 2.14 | 2.4 | 0.171 |
|  | Total | 1,362 | 24.641 | 156.22 | 6.15 | 4.97 | 3.48 | 2.81 | 0.186 |
| 2016 | Fire | 1,237 | 18.655 | 102.16 | 6.35 | 7.00 | 4.98 | 3.73 | 0.234 |
|  | No Fire | 1,388 | 27.194 | 168.43 | 5.69 | 4.97 | 3.21 | 2.46 | 0.180 |
|  | Total | 1,321 | 23.399 | 138.97 | 6.19 | 5.58 | 4.15 | 3.06 | 0.201 |

**Table S2.** Statistical results of the Generalized Estimation Equation (GEE) for the dependent variables mortality rate (M) and recruitment (R) in relation to fire predictor variables and monitoring period. χ² Wald: Chi-quadrad of Wald, DF: Degrees of freedom.

|  | **M** | | | **R** | | |
| --- | --- | --- | --- | --- | --- | --- |
|  | χ² Wald | DF | p-value | χ² Wald | DF | p-value |
| (Intercept) | 172,498 | 1 | 0,000 | 88,435 | 1 | 0,000 |
| Forest Fire | 11,584 | 1 | 0,001 | 0,182 | 1 | 0,670 |
| Interval | 116,085 | 4 | 0,000 | 40,209 | 4 | 0,000 |
| Forest Fire*Interval | 38,809 | 4 | 0,000 | 11,132 | 4 | 0,025 |

**Tabela S3.** Statistical results of the Generalized Estimation Equation (GEE) for the dependent variables Above-ground Biomass (AGB) and number of stems (NS) in relation to prediction variables forest fire (FF) and year of monitoring (Year).

|  | **AGB** | | | **NS** | | |
| --- | --- | --- | --- | --- | --- | --- |
|  | χ² Wald | DF | p-value | χ² Wald | DF | p-value |
| (Intercepto) | 103,960 | 1 | 0,000 | 8768,950 | 1 | 0,000 |
| FF | 6,220 | 1 | 0,013 | 3,034 | 1 | 0,082 |
| Year | 65,453 | 5 | 0,000 | 17,445 | 5 | 0,004 |
| FF*Year | 8,215 | 5 | 0,145 | 20,627 | 5 | 0,001 |

| 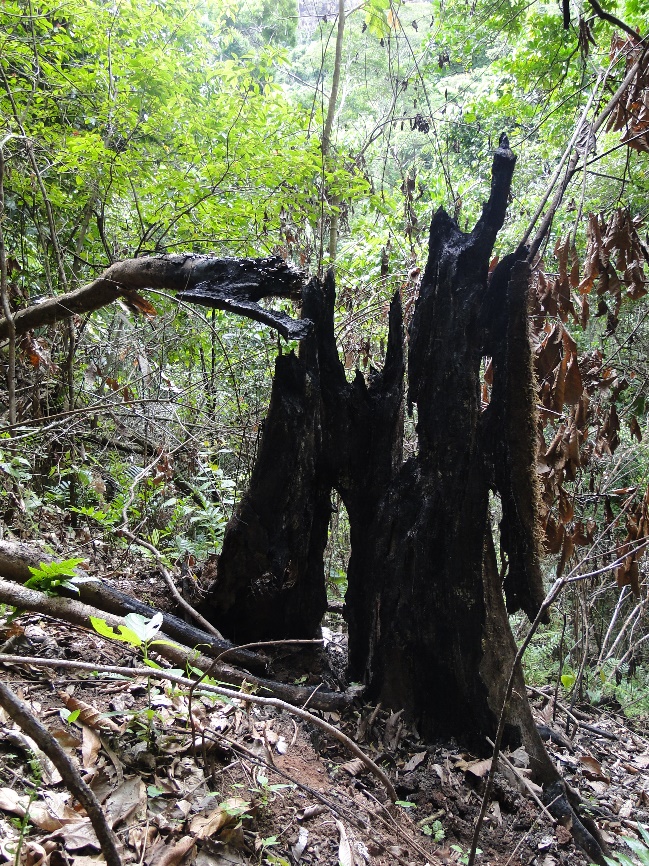 | 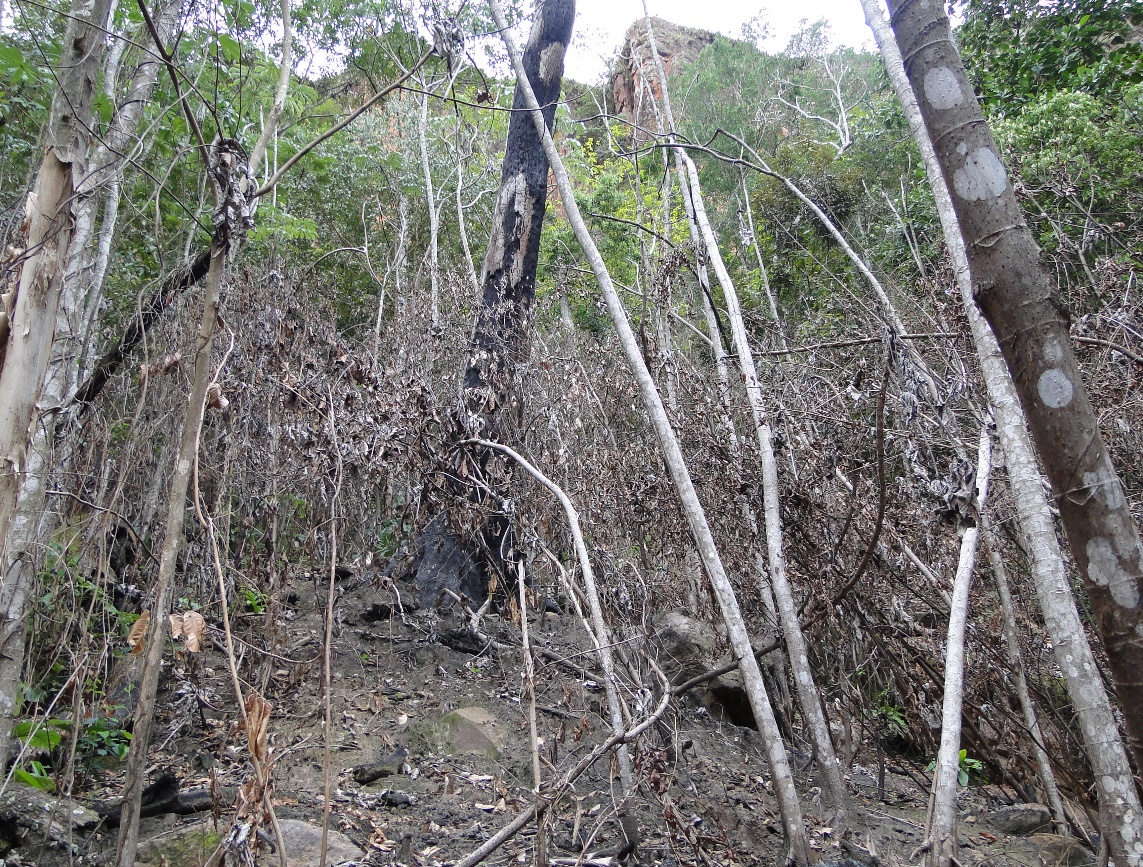 |
| --- | --- |

**Figure S1**: Evidence of the occurrence of the natural forest fire, registered in July 2010 in the Forest of Vale *Véu de Noiva* (FVVN) within the National Park of *Chapada dos Guimarães*, Brazil.
